# Depressive symptoms and academic achievement in UK adolescents: a cross-lagged analysis with genetic covariates

**DOI:** 10.1016/j.jad.2021.01.091

**Published:** 2021-04-01

**Authors:** José A. López-López, Alex S.F. Kwong, Liz Washbrook, Kate Tilling, Mina S. Fazel, Rebecca M. Pearson

**Affiliations:** 1 Department of Population Health Sciences, Bristol Medical School, University of Bristol (UK); 2 Centre for Academic Mental Health, University of Bristol (UK); 3 Department of Basic Psychology & Methodology, Faculty of Psychology, University of Murcia (Spain); 4 MRC Integrative Epidemiology Unit, University of Bristol (UK); 5 School of Education, University of Bristol (UK); 6 Centre for Multilevel Modelling, University of Bristol (UK); 7 Department of Psychiatry, University of Oxford (UK)

**Keywords:** adolescence, depression, academic achievement, cross-lagged, ALSPAC, polygenic risk scores

## Abstract

**Background:**

The relationship between adolescent depressive symptoms and academic achievement remains poorly understood. The aim of this study was to help clarify the nature and directionality of this association.

**Methods:**

We used a sample of 13,599 British adolescents (main sample of N=3,809 participants). We fitted cross-lagged panel models using four repeated measures of self-reported depressive symptoms and four measures of academic achievement based on British national records between 11-18 years, separately for male and female adolescents and considering polygenic risk scores (PRS) for educational attainment and depression, alongside other child and parental covariates.

**Results:**

We found evidence of an overall negative association that was stronger in boys (R=-0.21, 95% CI -0.31 to -0.11) than in girls (-0.13, -0.31 to 0.05). Higher depressive symptoms were associated with lower academic achievement at a later stage up to the end of compulsory education (16 years), when the direction of the association reversed, although girls with lower achievement also appeared vulnerable to depressive symptoms at previous stages. The genetic variables derived for this study showed stronger associations for academic achievement, but the PRS for depression also showed a negative association with academic achievement in girls. Child intelligence quotient and peer victimization also showed relevant associations.

**Limitations:**

Observational design, variation around measurement times, missing data.

**Conclusions:**

Depressive symptoms and academic achievement should be considered jointly when designing school-based programmes for children and adolescents, alongside gender, child ability and school experience. Including genetic information in research can help to disentangle average from time-varying effects.

## Highlights

• Adolescent depressive symptoms and academic achievement are negatively associated
• Depressive symptoms might affect later achievement in early adolescence (age 11-16)
• Poor results at the end of compulsory education (16 years) might affect later mood
• Genetic variables can now be derived, expect stronger associations with education
• Child IQ and peer victimization affect both depressive symptoms and academic results

## Introduction

The global burden of depression is considerable and adolescence is a key period in the development and presentation of first symptoms (Kwong, Manley, et al., 2019; Thapar et al., 2012). Furthermore, reoccurrence among individuals who have been depressed is common, hence the importance of understanding causal mechanisms that can be targets for prevention and early intervention strategies for children and young people (Davey & McGorry, 2019; Williams & Beidas, 2019) especially within universal settings such as education systems.

One of the key potentially modifiable pathways linking depression to longer term adversity is its association with academic achievement (Davies et al., 2018; Riglin et al., 2014). Academic achievement, and in particular marks, has been shown to influence future employment, health, and social functioning worldwide (Hale & Viner, 2018; Rodwell et al., 2018). The relationship between depressive symptoms and academic achievement in adolescence has drawn substantial attention (Fergusson & Woodward, 2002; Riglin et al., 2014; Veldman et al., 2014). However, this association remains poorly understood, with substantial uncertainty around its directionality, change over time and co-existence of anxiety and/or externalising symptoms (Fazel et al., 2014; Hale & Viner, 2018). It has been claimed that the relationship between depressive symptoms and academic achievement fades after adjusting for confounders (Fergusson & Woodward, 2002). Disentangling the role of depression in academic achievement has the potential to inform prevention and early intervention strategies aimed at young people to improve both depression and education (Davey & McGorry, 2019).

The association between depressive symptoms and academic achievement is best examined taking a longitudinal approach where repeated measures of each construct are collected. When multiple measures of both constructs at similar time points are available, they can be incorporated into a cross-lagged panel model. Cross-lagged analysis relates the level of depressive symptoms at a given time point to the change in academic achievement over a subsequent period (and *vice versa*), effectively controlling for simultaneity in the relationship between the two constructs at baseline.

Several cross-lagged analyses have examined the associations between depressive symptoms and academic achievement. Some of these studies have collected repeated measures of both internalising (which includes depressive symptoms) and externalising symptoms (such as behavioural problems), with the aim to isolate the potential contribution of each to academic performance and to test the so-called cascade hypotheses (Masten et al., 2005; Moilanen et al., 2010; Vaillancourt et al., 2013; Van Der Ende et al., 2016; Zhang et al., 2019). Results from these studies point to a negative correlation between externalising symptoms and later academic achievement, with lower achievement also being associated with higher depressive symptoms at a further point. Another set of studies focused on depressive symptoms and their associations with academic achievement during adolescence solely (Cole et al., 1996; Obradović et al., 2010), with important variations in the results reported across studies that suggest a complex and potentially bidirectional association that might be moderated by sex. In particular, it has been reported in previous studies that a bidirectional association might only be present in girls (Verboom et al., 2014), and that girls with low academic performance might be most vulnerable to depressive symptoms (Panayiotou & Humphrey, 2018; Pomerantz et al., 2002).

Some common limitations of previous studies are sample size (sometimes as small as 200 participants), few repeated measures (usually 2-3), long intervals between measurements (sometimes over 5 years), and a lack of normalized measures of academic achievement. These aspects might have precluded examination of potential sensitive periods such as the end of compulsory education. With regards to covariates, the cross-lagged analyses conducted so far in this field typically considered aspects such as parental education and socio-economic status, child ability and school experience. On the other hand, genetic differences among participants have not yet been considered in this context, which might explain why some key aspects of the association between depressive symptoms and academic achievement remain unclear.

### Use of polygenic risk scores

Studies examining the associations between depressive symptoms and academic achievement routinely consider potential psychosocial covariates (including parental socioeconomic position, parental educational level, quality of the relationships of the child with their peers) and psychological constructs such as child's intelligence quotient and parental mental health. Biological aspects, particularly genetic factors, have been suggested as potentially important when examining psychological processes (Steele et al., 2013), but have seldom been studied in this context until now – mostly due to a lack of available data in population studies. However, if depression and academic achievement share a common genetic background, analyses failing to account for this may be subject to confounding. For example, the same genetic variants may be linked to both risk of depression and poor academic achievement and this may create an observed link between the two observed variables even though one does not cause the other.

Recent developments in molecular biology provide an opportunity for novel analyses to incorporate variance explained by genetics into developmental studies. Specifically, genome-wide association studies (GWAS) have identified genetic variants associated with specific phenotypes, including depression (Howard, Adams, et al., 2019; Wray, Ripke, et al., 2018) and educational achievement (Lee et al., 2018; Okbay, Beauchamp, et al., 2016). Such genetic variants are known as single nucleotide polymorphisms (SNPs). SNPs from GWAS studies can be used to create polygenic risk scores (PRS) (Martin et al., 2019), which consist of weighted summary scores of the SNPs that measure an individual's liability to each trait. To illustrate the process, in this study we listed SNPs identified in the GWAS study of Howard and colleagues (2019), and used them to create a PRS for depression in each of our participants with genetic data available, so that a higher PRS represents higher genetic liability to depression. Despite the fact that most GWAS are conducted in adult populations, there is evidence that PRS for traits like depression are associated with depression in adolescence (Halldorsdottir, Piechaczek, et al., 2019; Rice, Riglin, et al., 2019), thus highlighting a strong genetic component during development. PRS can be added as covariates to statistical models in order to adjust for genetic factors relevant to each construct of interest as well as shared genetic variance justified by the finding of some overlapping genetic variants for depression and education (Wray et al., 2018). Importantly, incorporating PRS scores as covariates can also help disentangle timings and directionality of any association (Rice, Riglin, et al., 2019). This is because we know that any variance explained by genetic scores was assigned at conception and thus the influence of education or depression risk scores on later education or depression has a known direction and *vice versa*. This does not mean that PRS scores are deterministic, but instead can provide valuable insights to analyses aimed at disentangling timings and directions, which to our knowledge has not yet been illustrated in the context of cross-lagged panel models. Finally, evidence from large scale GWAS studies have shown a negative genetic correlation between depression and educational attainment, providing evidence of a relevant relationship at the genetic level (Wray, Ripke, et al., 2018).

### Aim and hypotheses

The aim of this paper was to clarify the nature of the relationship between depressive symptoms and academic achievement in adolescence. To this end, we included a large sample to get precise estimates separately for male and female adolescents, covered an extensive time frame to examine the nature of the relationships at different key periods, and considered standardised measures of academic achievement. To examine our hypotheses, we first conducted unadjusted analyses and then incorporated biological variables (namely PRS for depressive symptoms and academic achievement), family indicators (socioeconomic status and maternal education), and child variables (IQ and peer victimization). We hypothesized:

(1) A negative association of depressive symptoms with later academic achievement (Davies et al., 2018; Riglin et al., 2014; Sellers et al., 2019).
(2) A negative association of academic achievement with later depressive symptoms (Moilanen et al., 2010; Verboom et al., 2014).
(3) Stronger associations between depressive symptoms and academic achievement for girls (Verboom et al., 2014)
(4) Evidence for a sensitive period: poor academic achievement at the end of compulsory education (age 16 years) has important social implications and hence will show a stronger (negative) association with later depressive symptoms (Masten et al., 2005; Obradović et al., 2010).
(5) A positive association between the PRS for depression and observed depressive symptoms (Howard, Adams, et al., 2019; Rice, Riglin, et al., 2019; Wray, Ripke, et al., 2018).
(6) A positive association between the PRS for education and observed academic achievement (Lee et al., 2018).

## Method

### Participants: The Avon Longitudinal Study of Parents and Children (ALSPAC)

Pregnant women resident in Avon, UK, with expected dates of delivery 1st April 1991 to 31^st^ December 1992 were invited to take part in the study. The initial number of pregnancies enrolled is 14,541. Of these initial pregnancies, there was a total of 14,676 foetuses, resulting in 14,062 live births and 13,988 children who were alive at 1 year. When the oldest children were approximately 7 years, an attempt was made to bolster the initial sample with eligible cases who had failed to join the study originally. Further details of the sample can be found elsewhere (Boyd et al., 2013; Fraser et al., 2013). ALSPAC parents and children have been followed longitudinally, with mothers, mothers’ partners, and children providing data through postal questionnaires and clinic visits. Please note that the study website contains details of all the data is available through a fully searchable data dictionary and variable search tool (http://www.bristol.ac.uk/alspac/researchers/our-data/). Informed consent for the use of data collected via questionnaires and clinics was obtained from participants following the recommendations of the ALSPAC Ethics and Law Committee at the time. Individuals consented to participate in the study on the understanding that all measures would be used for research purposes only and not to inform decisions about their health.

### Measures

#### Depressive symptoms

The Short Mood and Feelings Questionnaire (SMFQ) is a self-reported questionnaire of 13 statements measuring depressive symptoms widely used for adolescent screening and monitoring purposes (Angold et al., 1996)**.** Individuals are asked to appraise each phrase as descriptive of their experiences ‘most of the time’, ‘sometimes’, or ‘not at all’ in the past two weeks. The total score ranges between 0 and 26, with higher scores indicating more depressive symptoms. We used four SMFQ measures around the ages of 11 (mean of 10.6, standard deviation of 0.26), 14 (M=13.8, SD=0.21), 16 (M=16.7, SD=0.24), and 18 (M=17.8, SD=0.40) years.

#### Academic achievement

ALSPAC has been linked to administrative records of individuals’ results on the National Pupil Database, which contains standardized assessments taken by state school pupils in England at ages 11, 14, 16 and 18. Although compulsory schooling for this sample ends at age 16, nationally, approximately 85% continue to further education in England (Department for Education, 2012).

**Scholastic Assessment Test at age 11**: We derived a continuous measure of achievement by averaging marks in standardized English, Maths and Science tests at the end of Year 6 (the final year of primary school) when pupils were a mean age of 11.2 (SD=0.32) years. For descriptive analyses (Table 1), a binary indicator captured whether the child achieved the “expected level” (National Curriculum Level 4) in all these three subjects.

**Table 1:** Descriptives, mean and SD for continuous variables, percentage for categorical variables)

	MAIN ANALYSIS SAMPLE			BROADER SAMPLE		
	Both sexes	Boys only	Girls only	Both sexes	Boys only	Girls only
Low SES	11.29%, N=3809	10.69%, N=1871	11.87%, N=1938	21.17%, N=12307	21.46%, N=6287	20.88%, N=6020
Mother finished school	85.72%, N=3809	85.14%, N=1871	86.27%, N=1938	81.72%, N=7435	81.88%, N=3715	81.56%, N=3720
Child IQ	105.8 (14.2), N=3809	106.1 (14.7), N=1871	105.4 (13.6), N=1938	103.2 (14.8), N=7317	103.2 (15.3), N=3627	103.2 (14.2), N=3690
Peer victimization [Md (Q1-Q3)]*	1 (0-3), N=3809	1 (0-3), N=1871	1 (0-2), N=1938	1 (0-3), N=7274	1 (0-3), N=3578	1 (0-3), N=3696
SMFQ score at 11 years	3.82 (3.35), N=3755	3.89 (3.26), N=1845	3.76 (3.43), N=1910	4.04 (3.51), N=7359	4.16 (3.45), N=3626	4.91 (3.56), N=3733
Achieved school goals at 11	84.58%, N=3346	82.34%, N=1625	86.69%, N=1721	70.91%, N=12133	67.78%, N=6108	74.07%, N=6025
SMFQ score at 14	4.83 (4.43), N=3158	4.02 (3.78), N=1518	5.58 (4.84), N=1640	4.92 (4.49), N=6015	4.09 (3.80), N=2939	5.71 (4.93), N=3076
Achieved school goals at 14	83.98%, N=2871	80.90%, N=1393	86.87%, N=1478	69.47%, N=10397	65.17%, N=5226	73.82%, N=5171
SMFQ score at 16	5.68 (5.44), N=2400	4.13 (4.35), N=1022	6.83 (5.86), N=1378	5.91 (5.64), N=4993	4.31 (4.58), N=2024	7.00 (6.02), N=2969
Achieved school goals at 16	68.63%, N=3271	64.38%, N=1586	72.64%, N=1224	50.33%, N=12024	45.19%, N=6065	55.56%, N=5959
SMFQ score at 18	6.34 (5.07), N=2269	5.43 (4.55), N=1001	7.05 (5.34), N=1268	6.58 (5.25), N=4495	5.63 (4.77), N=1907	7.29 (5.47), N=2588
Achieved school goals at 18	48.03%, N=3431	44.09%, N=1692	51.87%, N=1739	65.67%, N=6129	64.99%, N=2779	66.24%, N=3350

SES: socioeconomic status; IQ: intelligence quotient; SMFQ: Short Mood and Feelings Questionnaire

⁎ Peer victimization was summarized using median and first and third quartiles due to its skewed distribution

**Scholastic Assessment Test at age 14**: As at age 11, we averaged marks in standardized English, Maths and Science tests at the end of Year 9 (M=14.1, SD=0.31 years). For descriptive analyses (Table 1), a binary indicator captured whether the child achieved the “expected level” (National Curriculum Level 5) in all these three subjects.

**General Certificate of Secondary Education (GCSE)**: this is a series of exams taken at the end of compulsory education (M=16.1, SD=0.32 years in our sample), on a mixture of mandatory and optional subjects. We converted grades (A*-G) for each subject to points scores and took the total across the child's eight best subjects to generate total capped points scores. For descriptive analyses (Table 1), we derived a binary measure indicating whether the child achieved a pass in five or more exams including English and Maths.

**General Certificate of Education Advanced Level (A Level) and equivalent**: this is an optional exam normally taken between 16-18 years and considered as ‘pre-university’ level qualification. Participants in our sample had an average age of 17.1 years (SD=.49 years) at the start of the last recorded year. For all analyses, we used a binary indicator for whether an individual achieved a pass in three or more subjects. We imputed data for participants who either did not pursue an A Level or had no information on performance at this educational level (see statistical analyses section).

#### Covariates

**Genetic variables:** We used summary statistics from GWAS studies on depression (Howard, Adams, et al., 2019) and academic attainment (Lee et al., 2018) to select genetic variants associated with each phenotype. We computed a PRS for each construct to represent the genetic contribution to risk of depression and educational ability, using PRSice2 to create polygenic risk scores (Choi & O'Reilly, 2019). We used a 0.05 p-value threshold for loci selection in the main analyses, as this has been shown to explain the most amount of variance in previous studies (Wray, Ripke, et al., 2018). However, we also ran sensitivity analyses using thresholds of 0.5 and 0.005. Further details on PRS creation are presented elsewhere (Kwong, López-López, et al., 2019).

**Socioeconomic status (SES):** we used the earliest available information (usually from pregnancy) about mother's and partner's occupation to define a binary variable capturing low SES (unskilled, partly skilled, and skilled manual workers) and high SES (professional, managerial, technical, and skilled non-manual workers). We took the highest level of either parent to define SES at the family level.

**Maternal education:** at the child's eight-year clinic visit, mothers were asked whether they had attained a formal qualification at the end of compulsory school (GCSE or equivalent).

**Sex**: child biological sex at birth was used in this study.

**Intelligence Quotient:** child IQ was measured at the eight-year clinic visit using the Weschler Intelligence Scale for Children (Weschler et al., 1992), a well-validated instrument that provides scores of verbal and non-verbal IQ, which we averaged for the analyses.

**Peer victimization**: A modified version of the Bullying and Friendship Interview Schedule (Wolke et al., 2001) was administered at the child's ten-year clinic visit to assess self-reported peer victimization. Frequency of peer victimization was rated on a four‐point scale (0=never, 1=seldom, 2=frequently, 3=very frequently) across five different types of overt victimization (theft, threats or blackmail, physical violence, nasty names, nasty tricks), and four types of relational victimization (social exclusion, spreading lies or rumours, coercive behaviour, deliberately spoiling games). We used the total scores for the analyses.

### Statistical analyses

We fitted cross-lagged panel models within a structural equation modelling framework, using Mplus v8 (Muthén & Múthen, 1998). We included four standardized measures for depressive symptoms and academic achievement, using continuous scores (except for academic achievement at age 18, which was binary) and assuming the first measure for each construct as exogenous and the remaining measures as endogenous variables. Next, we added the covariates listed before as regressors of the age 11 person-centered variables (see below).

**Figure 1:**
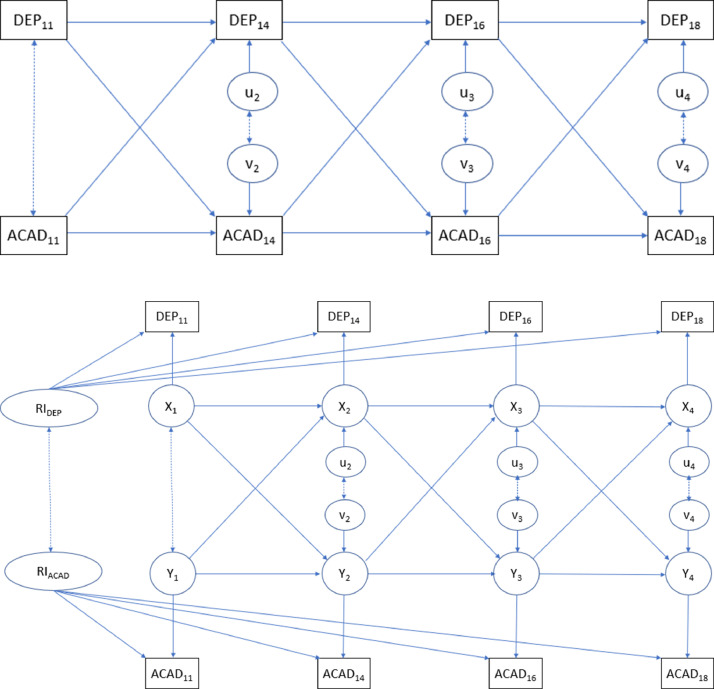
Cross-lagged panel models 1A. Standard cross-lagged panel model (CLPM) 1B. Cross-lagged panel model with random intercepts (RI-CLPM) DEP: depressive symptoms (at 11, 14, 16, and 18 years); ACAD: academic achievement (at 11, 14, 16, and 18 years); RI_DEP: random intercept for depressive symptoms; RI_ACAD: random intercept for academic achievement

We compared the fit of a standard cross-lagged panel model (CLPM, Figure 1A) and a more complex model including random intercepts for both constructs (RI-CLPM, Figure 1B), which decomposes the total variation into between-person and within-person variation (Hamaker et al., 2015). In Figure 1, the observed variables are represented by DEP_11_ to DEP_18_ (for depressive symptoms) and ACAD_11_ to ACAD_18_ (for academic achievement), whereas X_1_ to X_4_ and Y_1_ to Y_4_ represent within-person centered variables and between-person variation is introduced in the RI-CLPM model with the addition of RI_DEP_ and RI_ACAD_. We allowed for correlation between exogenous variables at wave one and between residuals (u and v in Figure 1) at subsequent waves. Regarding model fit indices, we considered Akaike's (AIC) and Bayesian information criteria (BIC), root mean square error of approximation (RMSEA), comparative fit index (CFI), Tucker-Lewis index (TLI), and standardized root mean residual (SRMR). Lower values for the AIC, BIC, RMSEA, and SRMR indices indicate better model fit, with RMSEA<0.05 and SRMR<0.10 often used as rule-of-thumb values to identify good approximate fit. Conversely, higher values are preferred for the CFI and TLI indices, with values over 0.95 commonly interpreted as reflecting good approximate fit (Kline, 2016).

Our main analysis sample included participants with at least one measure of depressive symptoms or academic attainment and full data on the covariates (N=3,809)*.* This is because the Mplus software does not allow imputation of covariates (only of dependent variables). The academic measures are essentially fully observed as these were taken from linked administrative data but, as is common in longitudinal surveys, there was missing questionnaire data. We dealt with missing data on academic achievement and depressive symptoms using full information maximum likelihood (FIML) estimation (Enders, 2010). In our main analyses we assigned a zero (goals not achieved) to participants who chose not to pursue further education after age 16 years. To assess the effect of the covariates we compared unadjusted and adjusted results, and also examined the standardized regression coefficients for each covariate. We performed and report these analyses separately for boys and girls.

We also considered a broader sample, namely those adolescents with at least one measure of depressive symptoms or academic attainment (N = 10,599), and compared the results of unadjusted models with those obtained using the main analysis sample of 3,809 participants. As a further sensitivity check we used FIML to impute academic records at age 18 for those with no education beyond compulsory schooling. This model effectively assigns a latent age 18 achievement outcome to those who left at 16, isolating effects of depressive symptoms on achievement conditional on participation and net of the drop-out decision. Furthermore, we spotted a spike in the distribution of SMFQ total scores at age 18, which was caused by a group of 183 participants responding ‘Sometimes true’ to every question in this and other related questionnaires. To test the robustness of our results, we ran another sensitivity analysis setting those participants as non-responders and using FIML to impute plausible values at that time point. Last, we fitted the model again using only participants with full information on all four measures of depressive symptoms and academic achievement (N = 1,554).

## Results

### Descriptive analyses

Descriptive characteristics for the whole sample and stratified by sex are presented in Table 1 for the main analysis and broader samples. With regards to the main analysis sample, around one in every ten participants came from low-SES families, with roughly 86% of mothers having completed school. Peer victimization scores were predominantly low (median of 1 for both sexes). Average scores for depressive symptoms were higher in females and showed an increase with time. Furthermore, reliability estimates of the SMFQ scores were α=.797 at age 11, α=.865 at age 14, α=.908 at age 16, and α=.897 at age 18. Regarding academic achievement, girls met the expected levels more often than boys at all compulsory stages. Compared to the broader sample, participants from the main analysis sample came from more affluent families, had slightly higher IQ scores, achieved school goals more frequently at compulsory stages and showed similar trends in depressive symptoms.

Table 2 shows the pair-wise correlations between PRS and the observed measures between ages 11 and 18. All coefficients were positive, suggesting a direct relationship between genotypes and phenotypes, and the magnitudes remained similar over time and across samples. Correlation estimates were substantially smaller between measures of depressive symptoms (range 0.043-0.094 in the main analysis sample) than between measures of academic achievement (range 0.28-0.35). Table 2 also shows a clear direct association between the PRS for education and child IQ (0.24 in the main sample). The PRS for education also exhibited smaller associations with maternal education and parental SES (0.157 and 0.128 in the main sample, respectively). Furthermore, the linear associations found between PRS and observed measures were substantially smaller than those yielded between repeated measures of the same construct (see Appendix 1).

**Table 2:** Pair-wise correlations between polygenic risk scores and other key variables

	Sample	Age 11	Age 14	Age 16	Age 18	Maternal education	Family SES	IQ
Depressive symptoms	MAIN (N = 1131)	.043	.089	.093	.094	.043	.018	-.052
	BROADER (N = 1156)	.049	.080	.089	.089	.038	.016	-.051
Academic achievement	MAIN (N = 2339)	.281	.335	.349	.267	.157	.128	.238
	BROADER (N = 2540)	.274	.337	.346	.273	.150	.129	.236

SES: socioeconomic status; IQ: intelligence quotient. The columns labelled “age 11”, “age 14”, “age 16”, and “age 18” refer to the repeated measures of each construct (depressive symptoms in the top rows and academic achievement in the bottom rows, respectively)

### Cross-lagged panel models

We used four repeated measures of each construct to fit both standard CLPM and RI-CLPM models. The model fit indices comparing both models supported use of the more complex RI-CLPM model, with an RMSEA of 0.053 (90% CI 0.044 to 0.062), CFI=0.990, TLI=0.97, and SRMR=0.021 in the main analysis sample (full results provided in Appendix 2). Therefore, for the remainder of this section we focus on five RI-CLPM models: M1 (unadjusted), M2 (including genetic covariates, the PRS), M3 (including family covariates, namely maternal education and parental socio-economic status), M4 (including child covariates, namely IQ and peer victimization), and M5 (fully adjusted).

Table 3 displays the coefficient estimates from the RI-CLPM models for boys. The unadjusted model yielded negative correlation estimates between achievement and depressive symptoms at the same time point at ages 11 and 14, which implies that better educational results were associated with lower depressive symptoms. Cross-lagged paths suggest that depressive symptoms were negatively associated with later academic achievement between ages 11 and 14 (-0.04, -0.08 to 0) and between ages 14 and 16 (-0.07, -0.12 to -0.02), with weak evidence of a positive association found between 16 and 18 years (0.39, -0.01 to 0.79). Furthermore, there was evidence of a positive association between academic achievement at age 14 and depressive symptoms at age 16 (0.19, 0.09 to 0.29). Moreover, coefficients linking measures of the same construct at different time points showed temporal persistence (e.g. direct relationships), which were stronger for academic achievement than for depressive symptoms except at ages 16 to 18. Comparison between the unadjusted and the adjusted models showed differences mainly at the cross-lagged paths between ages 16 and 18, with stronger evidence of a negative association between academic achievement at 16 and later depressive symptoms (-0.09, -0.17, -0.01 in the fully adjusted model) and a weaker but still positive association between depressive symptoms at 16 and academic achievement at 18 (0.07, 0, 0.14 in the fully adjusted model). Last, the unadjusted model provided evidence of a negative association between random intercepts (-0.21, -0.31, -0.11) that was also found in the adjusted models (in M2 and M5 the variance estimate was zero for the random intercept for academic achievement, hence the correlation between random intercepts was not estimable).

**Table 3:** Standardized estimates (with 95% confidence intervals) for RI-CLPM (cross-lagged panel models with random intercepts) models in boys (N = 1,871)

	Model 1: unadjusted	Model 2: genetic	Model 3: family	Model 4: child	Model 5: fully adjusted
***Auto-regressive paths***					
DEP14 on DEP11	0.12 (0.05, 0.19)	0.13 (0.05, 0.21)	0.13 (0.05, 0.21)	0.23 (0.16, 0.30)	0.24 (0.17, 0.31)
ACAD14 on ACAD11	0.81 (0.79, 0.83)	0.86 (0.84, 0.88)	0.85 (0.83, 0.87)	0.86 (0.84, 0.88)	0.86 (0.85, 0.87)
DEP16 on DEP14	0.14 (0.04, 0.24)	0.15 (0.05, 0.25)	0.15 (0.05, 0.25)	0.29 (0.20, 0.38)	0.3 (0.21, 0.39)
ACAD16 on ACAD14	0.74 (0.7, 0.78)	0.82 (0.79, 0.85)	0.8 (0.77, 0.83)	0.82 (0.8, 0.84)	0.82 (0.8, 0.84)
DEP18 on DEP16	0.30 (0.21, 0.39)	0.31 (0.22, 0.4)	0.31 (0.22, 0.4)	0.41 (0.33, 0.49)	0.42 (0.34, 0.5)
ACAD18 on ACAD16	0.27 (0.02, 0.52)	0.62 (0.56, 0.68)	0.58 (0.51, 0.65)	0.61 (0.57, 0.65)	0.62 (0.58, 0.66)
***Cross-lagged paths***					
DEP14 on ACAD11	-0.02 (-0.09, 0.05)	-0.01 (-0.09, 0.07)	-0.02 (-0.09, 0.05)	-0.02 (-0.08, 0.04)	0 (-0.06, 0.06)
ACAD14 on DEP11	-0.04 (-0.08, 0)	-0.03 (-0.06, 0)	-0.03 (-0.06, 0)	-0.03 (-0.06, 0)	-0.03 (-0.06, 0)
DEP16 on ACAD14	0.19 (0.09, 0.29)	0.12 (0.03, 0.21)	0.14 (0.04, 0.24)	0.1 (0.03, 0.17)	0.11 (0.04, 0.18)
ACAD16 on DEP14	-0.07 (-0.12, -0.02)	-0.05 (-0.09, -0.01)	-0.06 (-0.1, -0.02)	-0.06 (-0.10, -0.02)	-0.05 (-0.09, -0.01)
DEP18 on ACAD16	-0.07 (-0.17, 0.03)	-0.07 (-0.15, 0.01)	-0.07 (-0.16, 0.02)	-0.10 (-0.18, -0.02)	-0.09 (-0.17, -0.01)
ACAD18 on DEP16	0.39 (-0.01, 0.79)	0.1 (0.02, 0.18)	0.12 (0.03, 0.21)	0.06 (-0.01, 0.13)	0.07 (0, 0.14)
***Correlations***					
DEP11 with ACAD11	-0.15 (-0.21, -0.09)	-0.14 (-0.2, -0.08)	-0.15 (-0.21, -0.09)	-0.09 (-0.15, -0.03)	-0.08 (-0.14, -0.02)
DEP14 with ACAD14	-0.11 (-0.18, -0.04)	-0.12 (-0.19, -0.05)	-0.12 (-0.19, -0.05)	-0.12 (-0.18, -0.06)	-0.12 (-0.18, -0.06)
DEP16 with ACAD16	-0.05 (-0.14, 0.04)	-0.11 (-0.2, -0.02)	-0.1 (-0.19, -0.01)	-0.08 (-0.16, 0)	-0.08 (-0.16, 0)
DEP18 with ACAD18	0.01 (-0.19, 0.21)	0.01 (-0.07, 0.09)	0 (-0.08, 0.08)	-0.01 (-0.08, 0.06)	0.01 (-0.06, 0.08)
RI_DEP with RI_ACAD	-0.21 (-0.31, -0.11)	-	-0.25 (-0.48, -0.02)	-0.29 (-0.83, 0.25)	-

DEP: depressive symptoms (at 11, 14, 16, and 18 years); ACAD: academic achievement (at 11, 14, 16, and 18 years); RI_DEP: random intercept for depressive symptoms; RI_ACAD: random intercept for academic achievement

Results for girls are presented in Table 4. All models yielded negative correlations between different constructs measured at the same time point, and the unadjusted model shows a weak negative correlation between random intercepts of -0.13 (-0.31 to 0.05). This correlation could not be estimated in the adjusted models due to negative variance estimates in the random intercept for academic achievement that were truncated to zero. Regression coefficients within the same construct also showed direct relationships that were larger for academic achievement than for depressive symptoms across all measurement times. In the unadjusted model, the association between depressive symptoms and later academic achievement varied with age, with evidence of a negative association between14-16 years (-0.05, -0.09 to -0.01) which reversed between 16-18 years (0.09, 0.02 to 0.16). The unadjusted model provided inconclusive evidence on associations between academic achievement and later depressive symptoms, but the estimates from the adjusted models suggest negative associations around the later stages of compulsory education (in the fully adjusted model, these were estimated to be -0.07, -0.13 to -0.01 between 14-16 years and -0.05, -0.11 to 0.01 between 16-18 years). Note that these negative coefficient estimates suggest that higher achievement was associated with lower depressive symptoms at a later stage.

**Table 4:** Standardized estimates (with 95% confidence intervals) for RI-CLPM (cross-lagged panel models with random intercepts) models in girls (N = 1,938)

	Model 1: unadjusted	Model 2: genetic	Model 3: family	Model 4: child	Model 5: fully adjusted
***Auto-regressive paths***					
DEP14 on DEP11	0.04 (-0.04, 0.12)	0.04 (-0.04, 0.12)	0.03 (-0.05, 0.11)	0.14 (0.07, 0.21)	0.14 (0.07, 0.21)
ACAD14 on ACAD11	0.84 (0.8, 0.88)	0.86 (0.85, 0.87)	0.86 (0.85, 0.87)	0.86 (0.85, 0.87)	0.86 (0.85, 0.87)
DEP16 on DEP14	0.23 (0.16, 0.3)	0.23 (0.16, 0.3)	0.23 (0.16, 0.3)	0.31 (0.25, 0.37)	0.31 (0.25, 0.37)
ACAD16 on ACAD14	0.79 (0.72, 0.86)	0.82 (0.8, 0.84)	0.82 (0.8, 0.84)	0.82 (0.8, 0.84)	0.82 (0.8, 0.84)
DEP18 on DEP16	0.38 (0.32, 0.44)	0.38 (0.32, 0.44)	0.38 (0.32, 0.44)	0.44 (0.38, 0.5)	0.43 (0.37, 0.49)
ACAD18 on ACAD16	0.54 (0.33, 0.75)	0.63 (0.6, 0.66)	0.63 (0.6, 0.66)	0.62 (0.59, 0.65)	0.62 (0.59, 0.65)
***Cross-lagged paths***					
DEP14 on ACAD11	0.05 (-0.02, 0.12)	0.04 (-0.03, 0.11)	0.04 (-0.03, 0.11)	0.05 (-0.01, 0.11)	0.05 (-0.01, 0.11)
ACAD14 on DEP11	-0.01 (-0.04, 0.02)	-0.01 (-0.04, 0.02)	-0.01 (-0.04, 0.02)	-0.02 (-0.05, 0.01)	-0.02 (-0.05, 0.01)
DEP16 on ACAD14	-0.04 (-0.11, 0.03)	-0.05 (-0.12, 0.02)	-0.05 (-0.12, 0.02)	-0.07 (-0.13, -0.01)	-0.07 (-0.13, -0.01)
ACAD16 on DEP14	-0.05 (-0.09, -0.01)	-0.05 (-0.09, -0.01)	-0.05 (-0.09, -0.01)	-0.05 (-0.09, -0.01)	-0.05 (-0.09, -0.01)
DEP18 on ACAD16	-0.03 (-0.1, 0.04)	-0.05 (-0.12, 0.02)	-0.04 (-0.11, 0.03)	-0.05 (-0.11, 0.01)	-0.05 (-0.11, 0.01)
ACAD18 on DEP16	0.09 (0.02, 0.16)	0.08 (0.02, 0.14)	0.08 (0.02, 0.14)	0.07 (0.02, 0.12)	0.07 (0.02, 0.12)
***Correlations***					
DEP11 with ACAD11	-0.19 (-0.25, -0.13)	-0.19 (-0.25, -0.13)	-0.17 (-0.24, -0.1)	-0.08 (-0.14, -0.02)	-0.08 (-0.14, -0.02)
DEP14 with ACAD14	-0.02 (-0.08, 0.04)	-0.02 (-0.08, 0.04)	-0.02 (-0.08, 0.04)	-0.04 (-0.1, 0.02)	-0.03 (-0.09, 0.03)
DEP16 with ACAD16	-0.13 (-0.2, -0.06)	-0.13 (-0.2, -0.06)	-0.13 (-0.2, -0.06)	-0.12 (-0.19, -0.05)	-0.12 (-0.19, -0.05)
DEP18 with ACAD18	-0.05 (-0.12, 0.02)	-0.05 (-0.12, 0.02)	-0.05 (-0.12, 0.02)	-0.05 (-0.11, 0.01)	-0.05 (-0.11, 0.01)
RI_DEP with RI_ACAD	-0.13 (-0.31, 0.05)	-	-	-	-

DEP: depressive symptoms (at 11, 14, 16, and 18 years); ACAD: academic achievement (at 11, 14, 16, and 18 years); RI_DEP: random intercept for depressive symptoms; RI_ACAD: random intercept for academic achievement

Results presented in Tables 3 and 4 show some different results across models 1 to 5. The goodness of fit of the different models also showed some variations, with the fully adjusted model showing the lowest AIC and BIC values, which reflects a better fit to the data. Nonetheless, the more parsimonious model 1 (no covariates) was favoured by the CFI and SRMR indices (Appendix 3).

With regards to the specific associations found for each covariate, Table 5 shows the standardized regression coefficients, both from the partially adjusted models (models 2 to 4) and from the fully adjusted models (M5). There was no strong evidence of an association between the PRS for depression and the observed measures of depressive symptoms or academic achievement for boys. Conversely, there was evidence of a negative association with academic achievement in girls (B=-0.04, 95% CI -0.07 to -0.01 in M5). The PRS for education showed positive associations with academic achievement in both sexes (M5 estimates 0.12, 0.08 to 0.16 in boys and 0.10, 0.06 to 0.14 for girls). Maternal education was also directly associated with academic achievement in both sexes, but this association weakened after adding all covariates in M5 for girls (0.07, 0.03 to 0.11) and especially in boys (0.04, 0, 0.08). Moreover, fully adjusted models provided evidence of direct associations between family SES and academic achievement in boys (0.09, 0.05, 0.13) and girls (0.07, 0.04, 0.1), and even stronger positive associations between IQ and achievement (0.62, 0.59 to 0.65 in boys and 0.6, 0.57 to 0.63 in girls). Last, peer victimization yielded strong positive associations with depressive symptoms for both sexes (0.45, 0.41 to 0.49) as well as a negative association with academic achievement (-0.07, -0.10 to -0.04).

**Table 5:** Standardized coefficients (and 95% confidence intervals) for covariates included in RI-CLPM (cross-lagged panel models with random intercepts) models

	BOYS		GIRLS	
	DEPRESSIVE SYMPTOMS	ACADEMIC ACHIEVEMENT	DEPRESSIVE SYMPTOMS	ACADEMIC ACHIEVEMENT
PRS depression	M2: -0.01 (-0.05, 0.03) M5: -0.01 (-0.05, 0.03)	M2: -0.03 (-0.08, 0.02) M5: -0.01 (-0.04, 0.02)	M2: 0.02 (-0.03, 0.07) M5: -0.01 (-0.06, 0.04)	M2: -0.07 (-0.12, -0.02) M5: -0.04 (-0.07, -0.01)
PRS education	M2: -0.03 (-0.08, 0.02) M5: 0.01 (-0.04, 0.06)	M2: 0.31 (0.27, 0.35) M5: 0.12 (0.08, 0.16)	M2: -0.02 (-0.07, 0.03) M5: 0.03 (-0.02, 0.08)	M2: 0.29 (0.25, 0.33) M5: 0.1 (0.06, 0.14)
Maternal education	M3: -0.01 (-0.06, 0.04) M5: 0.02 (-0.03, 0.07)	M3: 0.16 (0.11, 0.21) M5: 0.04 (0, 0.08)	M3: -0.05 (-0.1, 0) M5: -0.01 (-0.06, 0.04)	M3: 0.22 (0.18, 0.26) M5: 0.07 (0.03, 0.11)
SES	M3: -0.02 (-0.07, 0.03) M5: 0 (-0.05, 0.05)	M3: 0.18 (0.13, 0.23) M5: 0.09 (0.05, 0.13)	M3: -0.04 (-0.09, 0.01) M5: -0.02 (-0.07, 0.03)	M3: 0.15 (0.1, 0.2) M5: 0.07 (0.04, 0.1)
IQ	M4: -0.08 (-0.12, -0.04) M5: -0.09 (-0.14, -0.04)	M4: 0.68 (0.65, 0.71) M5: 0.62 (0.59, 0.65)	M4: -0.11 (-0.16, -0.06) M5: -0.11 (-0.16, -0.06)	M4: 0.66 (0.63, 0.69) M5: 0.6 (0.57, 0.63)
Peer victimization	M4: 0.45 (0.41, 0.49) M5: 0.45 (0.41, 0.49)	M4: -0.08 (-0.12, -0.04) M5: -0.07 (-0.10, -0.04)	M4: 0.45 (0.41, 0.49) M5: 0.45 (0.41, 0.49)	M4: -0.07 (-0.11, -0.03) M5: -0.07 (-0.10, -0.04)

PRS: polygenic risk score; SES: socioeconomic status; IQ: intelligence quotient; M2: model adjusted for genetic covariates; M3: model adjusted for family covariates; M4: model adjusted for child covariates; M5: fully adjusted model

Sensitivity analyses based on the broader sample yielded similar trends to those found using the main analysis sample, but the cross-lagged paths showed some differences, including a larger negative effect of academic achievement at age 16 on depressive symptoms at age 18 (B=-0.1, -0.15 to -0.05 in the whole sample of 13,599 adolescents, see Appendix 4). Further sensitivity analyses are presented in Appendix 5, where FIML was used for participants with missing data on academic achievement at age 18 (this made no difference, since all 3,809 participants from the main analysis sample had full data for that variable), FIML was used again to estimate depressive symptoms at age 18 for participants with unusual response patterns (which resulted in a null association between academic achievement at age 16 and depressive symptoms at age 18). We found convergence problems when fitting the RI-CLPM model displayed in Figure 1B to participants with full information at all four time points (N = 1,554), so we fixed the random intercept variances to zero. Results showed stronger auto-regressive paths, particularly for depressive symptoms, and weaker associations regarding cross-lagged paths (Appendix 5). Last, Appendix 6 shows that the findings related to PRS variables were largely robust to the threshold used for loci selection, with evidence of a direct association between the PRS for education and academic achievement, as well as weak evidence of a negative association between the PRS for depression and academic achievement (analyses based on 3,809 girls and boys).

## Discussion

This study examined the association between depressive symptoms and academic achievement using four repeated measures obtained from a large British cohort between ages 11 and 18. We explored directionality separately for girls and boys by fitting cross-lagged models.

We found evidence of an overall negative association between depressive symptoms and academic achievement for both sexes. This is consistent with our first hypotheses and with previous research (Davies et al., 2018; Sellers et al., 2019). Of note, we found a stronger cross-sectional association at age 11 than at later time points, which differs from previous claims of a ‘constant’ relationship (Verboom et al., 2014).

With regards to directionality, our results point towards a bi-directional relationship. Depressive symptoms in early adolescence (11 and 14 years) were associated with worse academic achievement at later stages (14 and 16 years). Similar associations have been reported before in girls (Verboom et al., 2014), whereas our findings suggest a comparable relationship for both sexes. Conversely, we found evidence of lower academic achievement at age 14 associated with higher depressive symptoms at age 16 only for girls, whereas results for males were in the opposite direction. The vulnerability of poor performing girls to depression has been reported before (Panayiotou & Humphrey, 2018; Pomerantz et al., 2002) and might be linked to expectations, as nowadays it is well established that girls, on average, achieve better academic results than boys. For both sexes, fully adjusted models yielded negative associations between academic achievement at the end of compulsory education (age 16) and later depressive symptoms, which is consistent with the sensitive period hypothesis and with previous findings (Masten et al., 2005; Obradović et al., 2010) and highlights the importance of educational outcomes at this life stage.

Our hypothesis of a stronger association between depressive symptoms and academic achievement for girls was not supported by the data; in fact, the correlations between random intercepts from unadjusted models (Tables 3 and 4) suggests a slightly larger association for boys. We also found positive associations between depressive symptoms at age 16 and academic achievement at age 18. This finding was unexpected, as it goes in the opposite direction to what we observed in a previous study (López-López et al., 2020), and it might in fact reflect a positive association between depressive symptoms and the likelihood of remaining in (and completing) education beyond the minimum leaving age.

With regards to covariates, we observed a genetic link for the education PRS with academic achievement for both sexes, whereas the depression PRS showed smaller association with observed depressive symptoms (Table 5). These results might reflect differences in phenotype complexity and in the actual contribution of genetic variants in phenotypic expression. Apart from this, pair-wise correlations in Table 2 suggest that the PRS for education had greater predictive power than the PRS for depressive symptoms. Also, the correlation coefficients presented in Table 2 suggest that child IQ might mediate the association between the PRS for education and academic achievement, although this hypothesis would need to be confirmed or discarded in subsequent studies that specifically target pathways. Last, the finding of a negative association between the depression PRS and academic achievement in girls, as well as a (weaker) negative association between the PRS for education and depressive symptom in boys, point towards sex-specific directionalities of this association that could motivate further research.

Results for non-genetic covariates were comparable for both sexes. Child IQ showed a strong direct association with academic achievement and a small inverse association with depressive symptoms. Child IQ is known to be one of the main predictors of academic success (Lubinski, 2004), and has also been suggested as a protective factor for mental health problems in adolescence (Fergusson & Woodward, 2002; Pargas et al., 2010). Higher maternal education and family SES were associated with higher academic achievement. These are well established facts. SES has been found to be negatively associated with depressive symptoms (Johnson, Cohen, Dohrenwend, Link, & Brook,1999; Patalay & Fitzsimons, 2018), but we did not observe this pattern in our sample. Last, peer victimization yielded a large direct association with depressive symptoms, consistent with the literature (Chu, Fan, Lian, & Zhou, 2019; Evans, Smokowski, Rose, Mercado, & Marshall, 2019). Similar to Evans and colleagues, we found a small negative association between peer victimization and academic achievement.

### Implications for research and clinical practice

Our study provides further evidence of bidirectional, and potentially sex-specific, associations between depressive symptoms and academic achievement during adolescence. We achieved this by using a large, high-quality data resource. It is important that future studies continue to examine these associations, given the social implications of mental health and education and particularly at the start of adulthood. Some of the sex differences we found might be mediated by school attendance, which has been found to be associated with adolescent depression (Finning et al., 2019). Interestingly, another UK-based study found that absence rates were higher among boys, and that absenteeism was associated with poor educational performance (Woodfield et al., 2006).

For both sexes, we found that higher depressive symptoms are associated with poorer subsequent academic results up to year 11 (the end of compulsory education around age 16), when the directionality of this association reverses. Also, we found an association between poor academic results at age 14 and higher depressive symptoms at age 16 only in girls, which might be explained to some extent by the social/family expectations about girls performing well at school and the lower self-esteem of females likely to result following poor grades. This finding suggests that girls with low academic results at any age might benefit from interventions aimed at preventing depression. With regards to programmes intending to boost educational performance, our results showed that on average adolescents of any sex with depressive symptoms can be expected to have worse academic results in subsequent years.

A novelty of our study is the use of genetic data, indexed through by PRS. Our results suggest that it is now possible to create informative PRS to be used in psychological research, especially for education. Our findings largely confirmed the hypotheses of our study, and also showed some intriguing sex differences that could be examined further in coming years as the field of molecular biology continues to develop. We believe that use of genetic information can provide valuable insights about the nature of the complex relationship between depressive symptoms and academic achievement in adolescence. Nonetheless, we note that the current evidence is weaker for depressive symptoms, with smaller (and potentially underpowered) GWAS studies that combined data from patients identified using various clinical definitions of depression and different measurement instruments and formats.

Importantly, peer victimization also showed an association with both academic achievement and (especially) depressive symptoms. Given that peer relationships are modifiable, this highlights the importance of monitoring and supporting peer interactions promoting a healthy environment at schools.

The finding of a potentially bi-directional association is also relevant for clinicians and policy makers. Our results suggest that depressive symptoms might affect later achievement before the directionality reverses at the end of compulsory education. This is consistent with our hypotheses and provides a clear and actionable message to inform prevention and early intervention strategies for children and young people (Davey & McGorry, 2019; Williams & Beidas, 2019). In sum, our findings may be used in the design of public mental health interventions intended to impact positively on both areas.

It is unclear why the direction of association between depression and education appears to reverse after compulsory education. A positive association could be hypothesised through links between depression and perfectionism (Shafran & Mansell, 2001) as well as perfectionism and higher achievement (Madigan, 2019). It could be that within more specialized and self-selected studies perfectionism plays a greater role in both depression and achievement. It is important to note that this is a general population sample and that a different sample with higher clinical symptoms or depression requiring treatment may not show the same pattern.

We did not include anxiety or externalizing symptoms in our analyses. We acknowledge their importance in this context. However, it is problematic to assume that anxiety and/or externalizing symptoms have a unique role in the association between depressive symptoms and academic achievement. For instance, anxiety might cause both depression and academic failure for some children (e.g., it confounds the association of interest), but have a mediator role, or even be a common effect of depressive symptoms and academic results for other children. In this type of scenario, adjusting for anxiety would not be appropriate and would lead to misleading results: if anxiety is a mediator in the association of interest, adjusting for it would underestimate the magnitude of the association; conversely, if anxiety were a common effect of depression and educational results, then adjusting for it might result in an overestimation of the association between depression and academic achievement (this is sometimes referred to as “collider bias”, cf. Hernán and Robins, 2020). Because anxiety and externalizing symptoms do not fall into the category of “clear cut confounders”, we decided against including them in our models.

## Limitations

Some limitations of our study relate to the timings of the measurements. First, timings of the SMFQ measures do not exactly match academic measures. Appendix 7 provides the spread of lapses between both measures at each time point, which shows that most participants provided measurements of their depressive symptoms prior to doing their exams except for age 16. This suggests that the cross-lagged effects of depressive symptoms on later academic achievement might have been underestimated, as well as the potential impact of educational results at age 16 on depressive symptoms at age 18. Furthermore, adolescent mood is known to fluctuate, and therefore measurements taken at intervals of around two years might not reflect the full picture for all participants. Regarding educational measures, we took the most comprehensive measures of academic achievement at each educational stage. While English, Math and Science are the only subjects included at ages 11 and 14, some other relevant subjects were also available at ages 16 and 18, and we decided that those should be considered as well in our analyses.

Cross-lagged correlations stemming from observational longitudinal studies are prone to confounding and therefore do not provide as strong evidence as randomized controlled trials with regards to causal inference (Rogosa, 1980). We fitted several models adjusting for a range of genetic, child and family covariates and examined how this impacted on the results. Moreover, missing data assumptions are challenging for most longitudinal studies, especially when including academic measures beyond compulsory education. We addressed this by using FIML for most measures and performing a number of sensitivity analyses where the missing-at-random scenario was assumed.

Lastly, both depressive symptoms and academic achievement are complex phenomena that may be affected by factors beyond those included as covariates in our study. These include other psychiatric conditions like anxiety, attention deficit hyperactivity disorder, sleeping problems and substance abuse, as well as behaviours that might be more prevalent in one sex, such as gaming in boys (Weis & Cerankosky, 2010) and social media engagement through smartphone use in girls (Lee et al., 2020).

## Conclusion

We found some associations with potentially important clinical and social implications between depressive symptoms and academic achievement throughout adolescence. The pattern was bi-directional, with depressive symptoms correlating negatively with later educational achievement before the direction reversed at the end of compulsory education, and the trends remained similar after adjusting for genetic, child and family variables. Therefore, our study provides further support to the hypothesis that depressive symptoms and academic achievement affect each other, and should therefore inform the development of school-based mental health initiatives and the design of public mental health interventions intended to impact positively on both areas (Fazel et al., 2014). The impact of academic results at the end of compulsory education may be especially important.

## Author statement

JALL, RMP, MSF, LW, and KT conceived the project. JALL, RMP, KT, ASFK, and JK planned the statistical analyses. JALL and ASFK conducted the statistical analyses. JALL and RMP drafted a full version of the manuscript. All authors contributed to and approved the final version.

## Funding

This work was supported by the UK Economic and Social Research Council [grant number ES/P00881X/1] and the European Research Council under the European Union's Seventh Framework Programme grants 758813 (Mental Health Intergenerational Transmission [MHINT]) from the European Research Council Grant Agreements. The UK Medical Research Council and Wellcome (Grant ref: 217065/Z/19/Z) and the University of Bristol provide core support for ALSPAC. A comprehensive list of grants funding is available on the ALSPAC website (http://www.bristol.ac.uk/alspac/external/documents/grant-acknowledgements.pdf); This research was specifically funded by MRC (grant numbers G0701503/85179), Wellcome Trust and MRC (and 102215/Z/13/Z and 102215/2/13/2), and Wellcome Trust (WT088806). GWAS data was generated by Sample Logistics and Genotyping Facilities at Wellcome Sanger Institute and LabCorp (Laboratory Corporation of America) using support from 23andMe. The corresponding author had full access to all the data in the study and had final responsibility for the decision to submit for publication.

## Conflict of Interest

The authors declare no conflict of interest.

## Appendix Supplementary materials

Image, application 1
