# Supplementary material for: Depressive symptoms and academic achievement in UK adolescents: a cross-lagged analysis with genetic covariates
Source: J Affect Disord. 2021 Apr 1;284:104–13. doi: 10.1016/j.jad.2021.01.091 (PMC8105173; doi:10.1016/j.jad.2021.01.091)
Supplement: Supplementary file 1 [file mmc1.docx]

## Appendices

### Appendix 1. Pair-wise correlations between the variables included in the models

|  | dep11 | acad11 | dep14 | acad14 | dep16 | acad16 | dep18 | acad18 |
| --- | --- | --- | --- | --- | --- | --- | --- | --- |
| dep11 | 1 |  |  |  |  |  |  |  |
| acad11 | -0.0545 | 1 |  |  |  |  |  |  |
| dep14 | 0.6568 | -0.0484 | 1 |  |  |  |  |  |
| acad14 | -0.0648 | 0.5188 | -0.0530 | 1 |  |  |  |  |
| dep16 | 0.4145 | -0.1170 | 0.4754 | -0.1145 | 1 |  |  |  |
| acad16 | -0.0678 | 0.6333 | -0.0422 | 0.6615 | -0.118 | 1 |  |  |
| dep18 | 0.4284 | -0.0413 | 0.5005 | -0.0751 | 0.5033 | -0.0466 | 1 |  |
| acad18 | -0.0292 | 0.0685 | -0.0111 | 0.0102 | -0.0102 | 0.0264 | -0.0111 | 1 |

dep: depressive symptoms; acad: academic achievement

### Appendix 2. Goodness of fit of standard CLPM and RI-CLPM models

#### 2A. Main analysis sample (N = 3,809)

|  | AIC | BIC | RMSEA (90% CI) | CFI | TLI | SRMR |
| --- | --- | --- | --- | --- | --- | --- |
| CLPM | 49791 | 49990 | 0.078 (0.070, 0.086) | 0.974 | 0.940 | 0.040 |
| RI-CLPM | 49659 | 49877 | 0.064 (0.056, 0.074) | 0.987 | 0.959 | 0.021 |

CLPM: standard cross-lagged panel model; RI-CLPM: cross-lagged panel model with random intercepts; AIC: Akaike’s Information Criterion; BIC: Bayesian Information Criterion; RMSEA: Root Mean Square Error of Approximation; CFI: Comparative Fit Index; TLI: Tucker Lewis Index; SRMR: Standardized Root Mean Residual

#### 2B. Broader sample (N = 13,599)

|  | AIC | BIC | RMSEA (90% CI) | CFI | TLI | SRMR |
| --- | --- | --- | --- | --- | --- | --- |
| CLPM | 144813 | 145054 | 0.082 (0.078, 0.086) | 0.970 | 0.931 | 0.043 |
| RI-CLPM | 144104 | 144359 | 0.053 (0.048, 0.057) | 0.990 | 0.971 | 0.019 |

CLPM: standard cross-lagged panel model; RI-CLPM: cross-lagged panel model with random intercepts; AIC: Akaike’s Information Criterion; BIC: Bayesian Information Criterion; RMSEA: Root Mean Square Error of Approximation; CFI: Comparative Fit Index; TLI: Tucker Lewis Index; SRMR: Standardized Root Mean Residual

### Appendix 3. Goodness of fit of unadjusted and adjusted RI-CLPM models (N = 3,809)

|  | AIC | BIC | RMSEA (90% CI) | CFI | TLI | SRMR |
| --- | --- | --- | --- | --- | --- | --- |
| M1: Unadjusted | 49659 | 49877 | 0.064 (0.056, 0.074) | 0.987 | 0.959 | 0.021 |
| M2: Adjusted for genetic covariates | 49358 | 49595 | 0.064 (0.059, 0.070) | 0.969 | 0.939 | 0.036 |
| M3: Adjusted for family covariates | 49419 | 49662 | 0.060 (0.054, 0.066) | 0.974 | 0.946 | 0.029 |
| M4: Adjusted for child covariates | 46957 | 47200 | 0.073 (0.067, 0.079) | 0.969 | 0.936 | 0.031 |
| M5: Fully adjusted | 46794 | 47087 | 0.062 (0.058, 0.066) | 0.954 | 0.923 | 0.035 |

CLPM: standard cross-lagged panel model; RI-CLPM: cross-lagged panel model with random intercepts; AIC: Akaike’s Information Criterion; BIC: Bayesian Information Criterion; RMSEA: Root Mean Square Error of Approximation; CFI: Comparative Fit Index; TLI: Tucker Lewis Index; SRMR: Standardized Root Mean Residual

### Appendix 4. Results for broader sample

|  | Both sexes (N=13,599) | Boys only  (N = 6,895) | Girls only  (N = 6,698) |
| --- | --- | --- | --- |
| ***Auto-regressive paths*** | | | |
| DEP14 on DEP11 | 0.05 (0, 0.1) | 0.13 (0.07, 0.19) | 0.02 (-0.04, 0.08) |
| ACAD14 on ACAD11 | 0.85 (0.84, 0.86) | 0.85 (0.84, 0.86) | 0.85 (0.84, 0.86) |
| DEP16 on DEP14 | 0.24 (0.2, 0.28) | 0.17 (0.09, 0.25) | 0.21 (0.15, 0.27) |
| ACAD16 on ACAD14 | 0.76 (0.75, 0.77) | 0.77 (0.76, 0.78) | 0.74 (0.73, 0.75) |
| DEP18 on DEP16 | 0.37 (0.33, 0.41) | 0.29 (0.22, 0.36) | 0.36 (0.31, 0.41) |
| ACAD18 on ACAD16 | 0.89 (0.8, 0.98) | 0.7 (0.58, 0.82) | 0.95 (0.81, 1.09) |
| ***Cross-lagged paths*** | | | |
| DEP14 on ACAD11 | -0.03 (-0.07, 0.01) | -0.06 (-0.11, -0.01) | -0.02 (-0.07, 0.03) |
| ACAD14 on DEP11 | -0.03 (-0.05, -0.01) | -0.03 (-0.05, -0.01) | -0.03 (-0.06, 0) |
| DEP16 on ACAD14 | 0.03 (-0.02, 0.08) | 0.19 (0.12, 0.26) | -0.03 (-0.09, 0.03) |
| ACAD16 on DEP14 | -0.06 (-0.09, -0.03) | -0.09 (-0.13, -0.05) | -0.08 (-0.12, -0.04) |
| DEP18 on ACAD16 | -0.1 (-0.15, -0.05) | -0.12 (-0.2, -0.04) | -0.12 (-0.18, -0.06) |
| ACAD18 on DEP16 | 0.51 (0.37, 0.65) | 0.7 (0.61, 0.79) | 0.48 (0.17, 0.79) |
| ***Correlations*** | | | |
| DEP11 with ACAD11 | -0.23 (-0.26, -0.2) | -0.22 (-0.26, -0.18) | -0.25 (-0.29, -0.21) |
| DEP14 with ACAD14 | -0.06 (-0.1, -0.02) | -0.11 (-0.16, -0.06) | -0.06 (-0.11, -0.01) |
| DEP16 with ACAD16 | -0.08 (-0.13, -0.03) | -0.14 (-0.21, -0.07) | -0.13 (-0.18, -0.08) |
| EDUC18 with ACAD18 | - | - | - |
| RI_DEP with RI_ACAD | - | - | - |

DEP: depressive symptoms; ACAD: academic achievement; RI: random intercept; some correlations were not estimable due to a negative variance estimate (involving ACAD18) that was truncated to zero

### Appendix 5. Comparison between main results and different assumptions

Results in this table are based on the main analysis sample of 3,809 participants and compare the following analysis approaches:

- Main analysis (N = 3,809): participants who did not continue in education until age 18 have 0 in ACAD18, participants with unusual spike at DEP18 are included as usual.
- FIML for ACAD18 (N = 3,809): missing data at EDUC18 was handled using FIML.
- FIML for DEP18 (N = 3,809): participants with unusual pattern at dep18 were set to missing for this variable, using FIML to impute plausible values for this variable instead.
- Participants with complete data only (N = 1,554): restricted to participants with all four measures for depressive symptoms and academic achievement. The random intercept variances were truncated to zero to avoid convergence problems, hence the correlation between random intercepts was not estimable.

|  | Main analysis | FIML for EDUC18 | FIML for DEP18 | Complete data |
| --- | --- | --- | --- | --- |
| ***Auto-regressive paths*** | | | | |
| DEP14 on DEP11 | 0.06 (0, 0.12) | 0.06 (0, 0.12) | 0.06 (0, 0.12) | 0.29 (0.24, 0.34) |
| ACAD14 on ACAD11 | 0.85 (0.83, 0.87) | 0.85 (0.83, 0.87) | 0.81 (0.79, 0.83) | 0.84 (0.82, 0.86) |
| DEP16 on DEP14 | 0.25 (0.19, 0.31) | 0.25 (0.19, 0.31) | 0.25 (0.2, 0.3) | 0.40 (0.36, 0.44) |
| ACAD16 on ACAD14 | 0.8 (0.76, 0.84) | 0.80 (0.76, 0.84) | 0.74 (0.71, 0.77) | 0.81 (0.79, 0.83) |
| DEP18 on DEP16 | 0.4 (0.35, 0.45) | 0.40 (0.35, 0.45) | 0.41 (0.36, 0.46) | 0.52 (0.48, 0.56) |
| ACAD18 on ACAD16 | 0.67 (0.59, 0.75) | 0.67 (0.59, 0.75) | 0.34 (0.17, 0.51) | 0.51 (0.47, 0.55) |
| ***Cross-lagged paths*** | | | | |
| DEP14 on ACAD11 | 0.04 (-0.01, 0.09) | 0.04 (-0.01, 0.09) | 0.02 (-0.03, 0.07) | 0.05 (0, 0.1) |
| ACAD14 on DEP11 | -0.02 (-0.04, 0) | -0.02 (-0.04, 0) | -0.02 (-0.05, 0.01) | -0.01 (-0.04, 0.02) |
| DEP16 on ACAD14 | 0.02 (-0.04, 0.08) | 0.02 (-0.04, 0.08) | 0.04 (-0.02, 0.1) | -0.05 (-0.1, 0) |
| ACAD16 on DEP14 | -0.02 (-0.05, 0.01) | -0.02 (-0.05, 0.01) | -0.03 (-0.06, 0) | -0.01 (-0.04, 0.02) |
| DEP18 on ACAD16 | -0.05 (-0.11, 0.01) | -0.05 (-0.11, 0.01) | 0.01 (-0.05, 0.07) | -0.03 (-0.07, 0.01) |
| ACAD18 on DEP16 | 0.08 (0.02, 0.14) | 0.08 (0.02, 0.14) | 0.16 (0.03, 0.29) | 0.03 (-0.01, 0.07) |
| ***Correlations*** | | | | |
| DEP11 with ACAD11 | -0.16 (-0.21, -0.11) | -0.16 (-0.21, -0.11) | -0.17 (-0.21, -0.13) | -0.17 (-0.22, -0.12) |
| DEP14 with ACAD14 | -0.05 (-0.1, 0) | -0.05 (-0.10, 0) | -0.05 (-0.1, 0) | -0.07 (-0.12, -0.02) |
| DEP16 with ACAD16 | -0.04 (-0.09, 0.01) | -0.04 (-0.09, 0.01) | -0.04 (-0.1, 0.02) | -0.04 (-0.09, 0.01) |
| DEP18 with ACAD18 | -0.1 (-0.16, -0.04) | -0,10 (-0.16, -0.04) | -0.02 (-0.11, 0.07) | -0.05 (-0.1, 0) |
| RI_DEP with RI_ACAD | -0.25 (-0.44, -0.06) | -0.25 (-0.44, -0.06) | -0.15 (-0.22, -0.08) | - |

DEP: depressive symptoms; ACAD: academic achievement; FIML: full information maximum likelihood

### Appendix 6. Standardized coefficients (and 95% confidence intervals) for PRS according to different thresholds for loci selection (N = 3,809)

| PRS threshold | DEPRESSIVE SYMPTOMS | | ACADEMIC ACHIEVEMENT | |
| --- | --- | --- | --- | --- |
|  | PRS depression | PRS education | PRS depression | PRS education |
| p≤.5 | M2: 0.01 (-0.02, 0.04)  M5: 0 (-0.03, 0.03) | M2: 0.02 (-0.01, 0.05)  M5: 0.01 (-0.02, 0.04) | M2: 0.01 (-0.01, 0.03)  M5: -0.02 (-0.04, 0) | M2: 0 (-0.03, 0.03)  M5: 0.12 (0.09, 0.15) |
| p≤.05 | M2: 0 (-0.03, 0.03)  M5: -0.01 (-0.04, 0.02) | M2: 0.03 (0, 0.06)  M5: 0.02 (-0.01, 0.05) | M2: 0.01 (-0.01, 0.03)  M5: -0.03 (-0.05, -0.01) | M2: -0.01 (-0.03, 0.01)  M5: 0.11 (0.08, 0.14) |
| p≤.005 | M2: 0.02 (-0.01, 0.05)  M5: 0 (-0.03, 0.03) | M2: 0.03 (0, 0.06)  M5: 0.01 (-0.02, 0.04) | M2: 0 (-0.02, 0.02)  M5: 0.01 (-0.02, 0.04) | M2: -0.02 (-0.04, 0)  M5: 0.10 (0.07, 0.13) |

### Appendix 7. Lapses between measures of depressive symptoms and academic achievement

Values and percentiles in the table below summarize the number of months between measurements of both constructs at the same time point. Positive values indicate that depressive symptoms were measured prior to academic achievement.

| TIME POINT (YEARS) | Observations | Minimum | P_25_ | P_50_ | P_75_ | Maximum |
| --- | --- | --- | --- | --- | --- | --- |
| 11 | 7338 | -31 | 2 | 6 | 10 | 24 |
| 14 | 5960 | -31 | -3 | 2 | 7 | 20 |
| 16 | 4975 | -45 | -9 | -6 | -6 | 6 |
| 18 | 3341 | -46 | 0 | 6 | 10 | 25 |
